# Neuromuscular adaptations to auto-regulated velocity-based versus fixed percentage-based squat training in sprinters

**DOI:** 10.3389/fphys.2026.1757046

**Published:** 2026-01-22

**Authors:** Hanzhao Guo, Lingfeng Zhang, Zhanfei Zheng, Chang Liu, Feng Chen, Wenhai Wu

**Affiliations:** 1 College of Sports Science, Guangzhou Huali College, Guangzhou, Guangdong, China; 2 Department of Physical Education, Sichuan Electronic and Mechanic Vocational College, Mianyang, Sichuan, China; 3 Department of Sports Science, Wenzhou Medical University, Wenzhou, Zhejiang, China; 4 Longcheng Junior Middle School, Shenzhen, China; 5 College of Physical Education Science, Lingnan Normal University, Zhanjiang, China

**Keywords:** countermovement jump, load monitoring, percentage-based training, resistance training, velocity-based training

## Abstract

**Purpose:**

To compare auto-regulated velocity-based training (VBT) with traditional fixed percentage-based training (PBT) on neuromuscular performance in collegiate sprinters.

**Methods:**

Twenty resistance-trained males performed 6 weeks of back squat exercise 3 times per week. Both groups completed five sets of five repetitions with 3-min inter-set rest, matched for exercise selection and volume. The VBT group adjusted load based on real-time barbell velocity to maintain a target mean propulsive velocity of ∼0.54 m·s^-1^ (≈80% 1RM), whereas the PBT group trained with a fixed 80% of pre-intervention 1RM without further adjustment. Countermovement jump (CMJ), Standing long jump (SLJ), 20-m sprint times (T20-m), maximal strength (1RM back squat), and COD (T-test) were measured pre- and post-intervention.

**Results:**

Both groups significantly improved CMJ height (VBT: +7.8%, ES = 0.48; PBT: +6.7%, ES = 0.44), relative peak power output (VBT: +4.1%, ES = 0.85; PBT: +3.8%, ES = 0.35), SLJ performance (VBT: +1.0%, ES = 0.37; PBT: +1.8%, ES = 0.15), T20-m sprint times (VBT: −3.7%, ES = 1.30; PBT: −1.6%, ES = 0.51), maximal strength (VBT: +16.4%, ES = 1.57; PBT: +11.5%, ES = 0.94), and COD performance (VBT: −3.2%, ES = 0.54; PBT: −1.5%, ES = 0.39). VBT elicited significantly greater improvements than PBT in 1RM strength, T20-m, and COD performance (P < 0.05), whereas changes in CMJ and SLJ did not differ between groups (P > 0.05).

**Conclusion:**

Both training methods improved CMJ, SLJ, T20-m, 1RM back squat, and COD performance, but VBT may be slightly favorable for collegiate sprinters focusing on maximal strength, sprint performance, and COD compared to PBT.

## 1 Introduction

Sprinting requires an explosive power that is linked to maximum strength, acceleration, speed, and change of direction and relates directly to training effect and competition outcomes. Resistance training (RT) is recognized as an effective method for improving muscle hypertrophy, power output, and rate of force development (Schoenfeld et al., 2016). The specific adaptive response to RT is influenced by multiple factors that define the training variables, including loading magnitude, number of sets and repetitions, exercise type, and rest duration (Kraemer and Ratamess, 2004). In recent year, a common method to determine RT loads known as percentage-based training (PBT), prescribes relative submaximal loads from a one repetition maximum (1RM). This method is widely recognized in the literature and has demonstrated both validity and reliability across various populations (Rhea and Alderman, 2004). However, daily 1RM values fluctuate due to neuromuscular performance and training adaptation, decreasing with fatigue or increasing significantly within a few weeks, making their assessment time-consuming, especially in large groups (Padulo et al., 2012). Therefore, continued training based on an outdated 1RM may not optimize the neuromuscular stimuli required to maximize adaptation. For these reasons, alternative methods for prescribing training loads have been established.

With advancements in commercially available kinetic and kinematic measurement devices, it is now possible to provide instantaneous feedback on various training variables, such as movement velocity. Accordingly, recent literature has explored velocity-based training (VBT) as an alternative method for dynamically adjusting resistance training loads in real-time (Gonzalez-Badillo et al., 2014; Pareja-Blanco et al., 2017). By monitoring barbell velocity, VBT enables immediate load modifications, accounting for daily fluctuations in neuromuscular performance (Weakley et al., 2021). There are several benefits of monitoring velocity during resistance training. Firstly, receiving immediate feedback on velocity can encourage athletes to sustain maximal effort throughout their training (Weakley et al., 2019b; Weakley et al., 2020), which may promote greater adaptive responses (Weakley et al., 2019a). Secondly, velocity tracking enables the accurate determination of target velocity zones that correspond to specific training loads, thereby enhancing the precision and effectiveness of training prescriptions (Garcia-Ramos et al., 2019; Conceicao et al., 2016). Thirdly, because velocity remains relatively stable when measured against fixed absolute loads, any significant fluctuations in movement speed can serve as reliable indicators of either fatigue or gains in strength (Gonzalez-Badillo and Sanchez-Medina, 2010; Garcia-Ramos et al., 2018; Banyard et al., 2021). Previous studies have compared VBT with traditional percentage-based loading, generally reporting similar improvements in maximal strength and jump performance, while findings for sprint and change-of-direction outcomes appear more variable across protocols and populations (Liao et al., 2021; Banyard et al., 2021). Recent evidence syntheses further suggest that adaptations may depend on how VBT is operationalized (e.g., velocity targets and adjustment rules) and the extent to which effort/fatigue is monitored and reported (Li et al., 2025; Tutar et al., 2025). Consequently, this real-time velocity feedback and individualized adjustment capability may optimize training intensity and minimize excessive fatigue, providing a practical and highly specific approach to resistance training prescription.

However, much of the existing literature often limits its scope to isolated outcomes such as maximal strength (1RM) and vertical jump (CMJ), without thoroughly exploring its impact on broader athletic performance metrics (Marian et al., 2016; Jimenez-Reyes et al., 2017; Dorrell et al., 2020). While previous studies indicate that VBT may yield comparable or even superior adaptations compared to traditional training methods, typically with reduced overall training volume (Zhang et al., 2022; Held et al., 2022), comprehensive evaluations involving sprint speed, change-of-direction (COD) ability, and horizontal jump performance are scarce. Given the diverse neuromuscular demands associated with sprinting, it is essential to investigate and compare the effects of VBT and PBT across a broader range of performance outcomes.

Therefore, the aim of the present investigation was to compare the effects of velocity-based training (VBT) and percentage-based training (PBT) on neuromuscular performance in collegiate sprinters. Specifically, this study examined changes in 1RM, CMJ, 20-m sprint time (T20-m), COD, and standing long jump (SLJ) to provide a more comprehensive evaluation of the training adaptations associated with each method.

## 2 Materials and methods

### 2.1 Participants

An *a priori* power analysis (α = 0.05, 1-β = 0.8) was performed using G*Power (Version 3.1.9.7) for the group × time interaction in a two-way repeated-measures ANOVA, assuming a moderate effect size (f = 0.30; ≈ d 0.5), which indicated a minimum total sample size of 18. To account for potential drop-outs (∼20%), twenty well-trained male sprinters (age: 21 ± 2.4 years, height: 177.8 ± 4.87 cm, body mass: 67.4 ± 4.5 kg) were recruited. All participants were university track-and-field sprinters and competed in 100–200 m intercollegiate events during the most recent season. Participants reported 3 years of resistance training experience (2 sessions/week) and were accustomed to performing the back squat, with a minimum 1RM of 1.5 times their body mass. Following familiarization and baseline testing, participants were randomized (1:1) to VBT or PBT using a computer-generated sequence with concealed allocation (opaque, sealed envelopes). All subjects were injury-free for at least 6 months before this study and did not complete any strenuous exercise in the 48 h before the start of the investigation. The study was approved by the *Wenzhou Medical University Review Board for Human Participants* (no. 2024078) and complied with the principles of the *Declaration of Helsinki*, with written informed consent obtained from participants.

### 2.2 Study design

A randomised controlled design was employed to investigate the effects of two conditions executed with different resistance-training protocols on physical performance. Following familiarization and pre-testing, participants were randomly assigned to either an VBT (n = 10) or PBT (n = 10) group. Participants completed intervention sessions three times per week over a 6-week period on non-consecutive days, with 72 h between the final training session and post-intervention testing to minimize residual fatigue. During the intervention, participants were instructed to maintain their habitual sprint/technical training routines and to refrain from any additional lower-limb resistance training beyond the prescribed program. Countermovement jump (CMJ) height, relative peak power output, standing long jump, one-repetition maximum (1RM) barbell back squat, T-test and 20-m sprint performance were taken before and after intervention. All tests were performed at least 48 h Pre/Post the most recent training session. Testing and training sessions were consistently performed at the same location, supervised directly by the lead investigator. Environmental conditions (approximately 20 °C and 60% humidity) and testing times (±1 h) were carefully controlled and standardized for each participant.

### 2.3 Resistance training program

The resistance training (RT) program consisted solely of the full squat (SQ) exercise performed on a Smith machine (Matrix Fitness, Aura Series, United States). Participants trained three times per week (Monday–Wednesday–Friday) for six consecutive weeks. Each session began with a standardized warm-up, including 5 min of aerobic exercise followed by dynamic stretches, and two warm-up sets at 50% and 70% of the training load. For the PBT group, loads were fixed at 80% 1RM, with participants performing five sets of five repetitions, maintaining a controlled eccentric (∼2 s) and explosive concentric phase, and 3 min of rest between sets. For the VBT group, training loads were adjusted session-by-session using mean concentric velocity (MCV) targets (Banyard et al., 2021; Gonzalez-Badillo and Sanchez-Medina, 2010). Specifically, if MCV differed by ±0.06 m·s^-1^ from the target velocity of 0.54 m·s^-1^, loads were adjusted by ±5%; if the difference was ±0.12 m·s^-1^ or greater, loads were adjusted by ±10%. All velocities were recorded using a linear position transducer (GymAware, Kinetic Performance Technology, Canberra, Australia) sampling at 50 Hz. All sessions were conducted at a consistent time (±1 h) in a laboratory under direct supervision. Verbal encouragement was provided throughout each session. Participants’ adherence was carefully monitored, with all participants completing at least 90% of scheduled training sessions.

#### 2.3.1 One-repetition maximum testing

Participants performed the 1RM test on a Smith machine following the National Strength and Conditioning Association (NSCA) guidelines (Haff and Triplett, 2016). Prior to testing, they completed a general warm-up consisting of 5 min of cycling on an ergometer (Power Max VIII; Konami Corporation, Tokyo, Japan), followed by dynamic stretching. A specific warm-up was then performed, including five repetitions at ∼50% of the estimated 1RM and three repetitions at ∼70% of the estimated 1RM. Subsequent attempts consisted of single repetitions with progressively increased loads (5%–10% increments for lower-body exercises and 2.5%–5% increments for upper-body exercises) until failure to complete a full range of motion. A metronome was used to maintain the speed of all squat repetitions at a pace of 2 s for the eccentric phase and 1 s for concentric phase (without jumping). Each participant 1RM was determined within five attempts, with a 3-min rest between trials. All tests were supervised by certified strength and conditioning specialists to ensure proper technique and safety.

#### 2.3.2 Countermovement jumps

For the CMJ, Participants were instructed to stand with their feet shoulder-width apart on a force platform (model 9260AA, Kistler Corporation, Switzerland) with their hands placed on their hips to avoid arm swings. The test began with the participant performing a preparatory squat by flexing the knees to approximately 90°, followed by a rapid extension of the hips and knees to jump as high as possible. Participants were given standardized verbal encouragement to maximize performance during each trial. A total of three CMJ trials were completed, with a 60-s rest period between each attempt to ensure adequate recovery. The highest jump height recorded was used for further analysis. Vertical Jump Height (VJH) was calculated the flight time from the initiation of the jump to landing. The relative peak power output (RPP) was determined by the force-time curve generated by the force platform during the jump, and normalized to body weight. All mechanical jump parameters were computed using MARS software (version 4.0; Kistler Instruments Corp, Switzerland).

#### 2.3.3 20-m sprint testing

Participants performed three 20-m sprints on an indoor running track, with a 2-min rest between sprints. Sprint time were measured using two electronic timing gates (Smart Speed Systems, Coopers Plains, QLD, Australia), which were fixed at 0 and 20-m at hip level. A standing position, with the self-selected lead-off foot placed 1 m behind the first timing gate, was used. The best record in T20 time was use for further analysis.

#### 2.3.4 Change of direction ability (COD)

**FIGURE 1:**
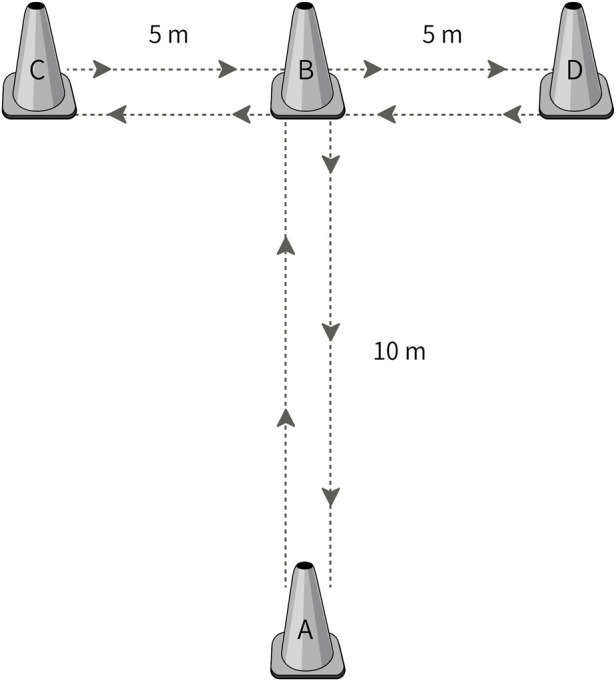
T-test procedure.

To assess the change of direction (COD) ability, the T-test (Figure 1) was used according to procedures by Pauole et al. (2000). The test was conducted on a non-slip surface, with participants wearing standardized athletic footwear. The course was marked with four cones arranged in a “T” shape: one starting cone (A), two lateral cones (C and D) 5 m apart, and one forward cone (B) positioned 10 m from the starting cone. Participants began in a standing position with their feet behind the starting cone. Upon receiving a verbal cue, they sprinted forward 10 m to touch cone B, then shuffled 5 m to the left to touch cone C, immediately changed direction to shuffle 10 m to the right to touch cone D, shuffled 5 m back to cone B, and finally backpedaled to the starting position (cone A). The total time to complete the course was recorded using electronic timing gates (Smart Speed Systems, Coopers Plains, QLD, Australia) positioned at the start and finish line at a height of 1.0 m. Participants completed three trials with at least 2 min of passive rest between attempts. The best times for the T-Test was used for further analysis.

#### 2.3.5 Standing long jump (SLJ)

During the SLJ test, participants stood with their feet shoulder-width apart behind a marked starting line. On the command of “ready, set, go”, they executed a rapid countermovement, involving simultaneous knee flexion and arm swing, before explosively jumping forward as far as possible. The horizontal distance between the starting line and the heel of the rear foot was recorded using a tape measure to the nearest 1-cm. Participants completed three maximal trials, with a 1-s rest between attempts. The best performance was recorded for further analysis.

### 2.4 Statistical analyses

Values are expressed as means ± SD. Test-retest absolute reliability was assessed using the CV, whereas relative reliability was calculated using the intraclass correlation coefficient (ICC) with a 95% CI, using the two-way mixed model. The normality of distribution of the variables in the Pre-test and the homogeneity of variance across groups (VBT vs. PBT) were verified using the Shapiro-Wilk test and Levene’s test, respectively. Independent-samples t-tests were conducted to assess the intergroup differences based on percentage changes from pre-to post-training. Two-way mixed (between-within) ANOVA, with Bonferroni *post hoc* comparisons, using one inter-factor (VBT vs. PBT) and one intra-factor (pre-vs. post-training), were conducted to examine the differences across all movements and jump protocols between groups. In addition, effect sizes (ES) were calculated using *Hedge’s g* on the pooled SD (Hedges and Olkin, 1985). Dependent variables were expressed as percent change (%) from pre-intervention. All statistical analyses were performed in SPSS (Version 29.0, IBM SPSS Statistical Software). Statistical significance was set at *p* ≤ 0.05.

## 3 Results

The main effect of time was observed for CMJ height and relative peak power output, standing long jump, 20-m sprint time, 1RM back squat and T-test time (all P ≤ 0.048), while a significant condition × time interaction was only found for 1RM back squat (P = 0.021).

**FIGURE 2:**
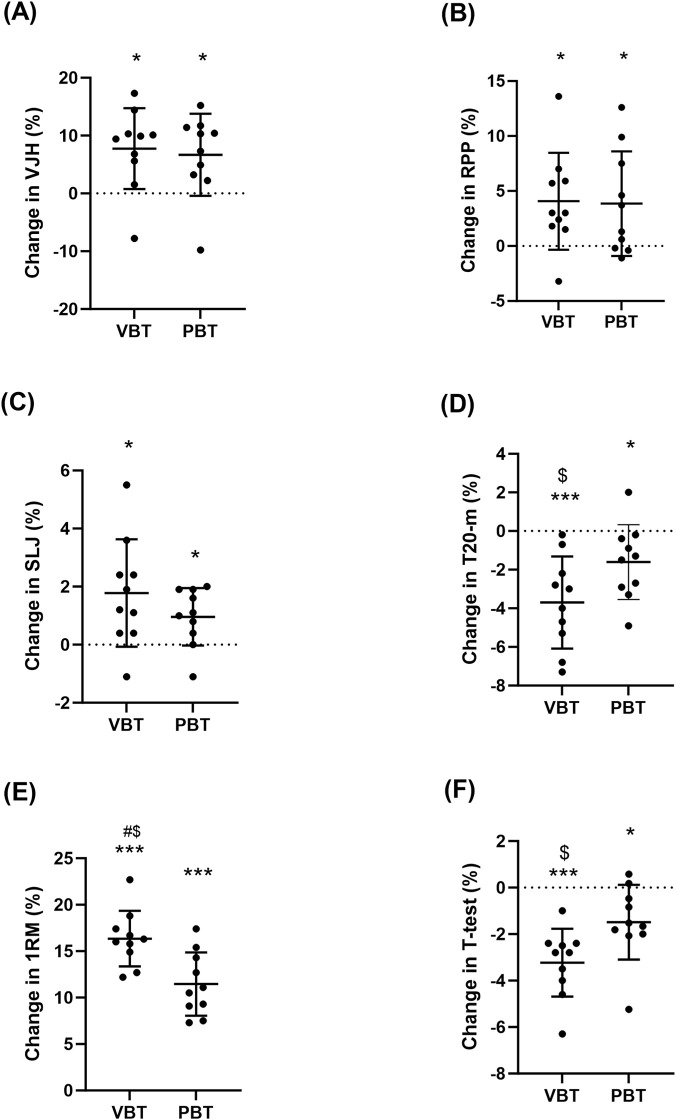
Individual and average percent change in selected neuromuscular performance variables: CMJ vertical jump height **(A)**, CMJ relative peak power output **(B)**, SLJ **(C)**, 20-m sprint time **(D)**, 1RM **(E)**, and T-test **(F)** for VBT and PBT groups. Statistically significant “time × condition” interaction: #p < 0.05. Statistically significant differences with respect to PBT $p < 0.05. Intra-group significant differences from Pre to Post: *p < 0.05, **p < 0.01, ***p < 0.001.

Participants exhibited increased CMJ height in both VBT (49.3 [7.2] vs. 53.0 [6.8] cm; +7.8% [6.3%]; *P* = 0.019; ES = 0.48) and PBT (45.4 [6.6] vs. 48.2 [5.7] cm; +6.7% [6.7%]; *P* = 0.017; ES = 0.44) from pretests to posttests (Table 1; Figure 2A). Compared with pretests, CMJ relative peak power output increased after VBT (62.4 [4.6] vs. 67.9 [7.3] W/kg; +4.1% [4.2%]; *p* = 0.026; ES = 0.85) and PBT (60.4 [6.2] vs. 62.6 [5.5] W/kg; +3.8% [4.5%]; *p* = 0.028; ES = 0.35; Table 1; Figure 2B). Both VBT (2.77 [0.12] vs. 2.82 [0.13] m; +1.0% [0.9%]; *p* = 0.015; ES = 0.37) and PBT (2.66 [0.13] vs. 2.68 [0.12] m; +1.8% [1.8%]; *p* = 0.014; ES = 0.15) resulted in standing long jump performance from pretests to posttests (Table 1; Figure 2C). From pretests to posttests, 20-m sprint times improved for both VBT (3.03 [0.07] vs. 2.92 [0.09] s; −3.7% [2.2%]; *p* = 0.027; ES = 1.30) and PBT (3.01 [0.09] vs. 2.96 [0.09] s; −1.6% [1.8%]; *p* < 0.001; ES = 0.51). When comparing percentage changes, VBT showed a significantly greater reduction in sprint times compared to PBT (*p* = 0.045; d = 0.96; Table 1; Figure 2D). Compared with pretests, 1 RM back squat increased after VBT (121.8 [11.4] vs. 141.5 [11.8] kg; 16.4% [2.7%]; *p* < 0.001; ES = 1.57) and PBT (126.3 [13.2] vs. 140.8 [15.3] kg; 11.5% [3.2%]; *p* < 0.001; ES = 0.94). When comparing percentage changes, VBT showed a significantly higher in 1RM back squat compared to PBT (*p* = 0.003; d = 1.52; Table 1; Figure 2E). Finally, participants exhibited increased T-test times after both VBT (10.37 [0.58] vs. 10.03 [0.59] s; −3.2% [1.3%]; *p* < 0.001; ES = 0.54) and PBT (10.26 [0.43] vs. 10.10 [0.33] s; −1.5% [1.5%]; *p* = 0.020; ES = 0.54) compared with pretests. When comparing percentage changes, VBT showed a significantly greater reduction in T-test times compared to PBT (*p* = 0.026; d = 1.16; Table 1; Figure 2F).

**TABLE 1:** Descriptive characteristics (mean ± SD) and effect sizes of VBT and PBT training groups, pre-training to post-training.

	VBT				PBT			
	Pre	Post	%△	ES	Pre	Post	%△	Es
CMJ height (cm)	49.3 ± 7.2	53.0 ± 6.8*	7.8%	0.48	45.4 ± 6.6	48.2 ± 5.7*	6.7%	0.44
CMJ relative peak power output (w/kg)	62.4 ± 4.6	67.9 ± 7.3*	4.1%	0.85	60.4 ± 6.2	62.6 ± 5.5*	3.8%	0.35
Standing long jump (m)	2.77 ± 0.12	2.82 ± 0.13*	1.8%	0.37	2.66 ± 0.13	2.68 ± 0.12*	1.0%	0.15
20-m sprint (s)	3.03 ± 0.07	2.92 ± 0.09***	−3.7% $	1.30	3.01 ± 0.09	2.96 ± 0.09*	−1.6%	0.51
1RM back squat (kg) #	121.8 ± 11.4	141.5 ± 11.8***	16.4% $	1.57	126.3 ± 13.2	140.8 ± 15.3***	11.5%	0.94
T-test (s)	10.37 ± 0.58	10.03 ± 0.59***	−3.2% $	0.54	10.26 ± 0.43	10.10 ± 0.33*	−1.5%	0.39

Statistically significant “time × condition” interaction: #p < 0.05. Statistically significant differences with respect to PBT.

$p < 0.05. Intra-group significant differences from Pre to Post: *p < 0.05, **p < 0.01, ***p < 0.001. Abbreviations: Pre: initial assessment; Post: final assessment; ES: intra-group effect size (Hedge’s g); 1 RM: one-repetition maximum; CMJ: countermovement jump; VBT: velocity-based training; PBT: percentage-based training.

## 4 Discussion

The aim of this study was to compare the effect of VBT and PBT over a 6-week resistance training intervention on neuromuscular performance variables in collegiate sprinters. The main finding was that both VBT and PBT significantly improved CMJ, SLJ, 20-m sprint times, 1RM back squat, and COD (T-test), confirming that both training approaches effectively enhance key aspects of athletic performance. However, VBT demonstrated significantly greater improvements in 1RM, sprint performance, and T-test compared to PBT. The use of real-time velocity feedback allows athletes to dynamically adjust their training intensity based on daily fluctuations in performance capacity, which may explain the superior outcomes observed in VBT groups in maximal strength and explosive performance measures.

In the present study, both the VBT and PBT groups significantly improved CMJ height and relative peak power output following a 6-week training intervention. Specifically, CMJ height increased by 7.8% (ES = 0.48) in the VBT group and 6.7% (ES = 0.44) in the PBT group. Similarly, CMJ relative peak power output increased by 4.1% (ES = 0.85) and 3.8% (ES = 0.35), respectively. These improvements are consistent with previous research demonstrating that structured resistance training interventions effectively enhance lower-limb explosive power by positively influencing neuromuscular factors such as increased motor unit recruitment, improved inter- and intra-muscular coordination, and enhanced stretch-shortening cycle (SSC) efficiency (Pareja-Blanco et al., 2017; Aagaard et al., 2002; Tillin and Folland, 2014). The slightly superior effect size observed in the VBT group may be attributed to the individualized nature of load prescription provided by real-time velocity feedback. According to (Gonzalez-Badillo et al., 2014), training at individually tailored velocities allows athletes to consistently train closer to their optimal power zone, potentially leading to greater improvements in explosive strength. Moreover, (Weakley et al., 2021), suggested that instantaneous velocity feedback during resistance training could enhance athlete motivation, encouraging maximal intent and effort throughout each repetition. Consequently, this elevated neuromuscular intent likely translates into greater improvements in explosive power-related performances, such as CMJ. Collectively, our findings suggest that while both VBT and PBT modalities effectively improve explosive performance in trained collegiate sprinters, the real-time load adjustments and heightened motivation facilitated by velocity feedback might offer slight but practically meaningful advantages in enhancing vertical jump performance and peak power output.

Beyond explosive power, substantial gains in maximal squat strength (1RM) were also observed in both groups, Notably, the VBT group showed a 16.4% increase (ES = 1.57), while the PBT group improved by 11.5% (ES = 0.94), with the VBT group achieving significantly greater strength gains compared to the PBT group. This finding aligns closely with previous studies indicating that VBT tends to result in superior maximal strength outcomes compared to PBT (Banyard et al., 2021; Gonzalez-Badillo and Sanchez-Medina, 2010). The strength improvements observed in both the VBT and PBT groups can be attributed to several physiological mechanisms induced by structured resistance training. Firstly, both methods likely enhance neuromuscular efficiency through increased motor unit recruitment, synchronization, and firing rate, contributing significantly to maximal force output adaptations (Suchomel et al., 2016). Additionally, repeated exposure to heavy loads, as employed in the PBT protocol, promotes adaptations in muscular strength by inducing mechanical stress that stimulates both neural adaptations and muscle hypertrophy, thereby elevating the force-generating capacity of muscle fibers over time (Schoenfeld et al., 2016; Kraemer and Ratamess, 2004). A plausible explanation for the greater strength improvements observed in the VBT group involves the individualized load adjustments enabled by velocity monitoring. Specifically, by continuously tracking lifting velocity, athletes can train consistently near their optimal intensity, accurately reflecting daily fluctuations in neuromuscular readiness (Weakley et al., 2021). In contrast, the fixed loads prescribed in PBT protocols may not effectively capture short-term fluctuations in strength levels, potentially leading to suboptimal stimuli during some training sessions (Pareja-Blanco et al., 2017). Moreover, individualized velocity-based adjustments can reduce unnecessary fatigue accumulation, thereby maintaining higher quality training stimuli throughout the intervention period, which could enhance maximal strength adaptations (Gonzalez-Badillo et al., 2014).

In addition to improvements in strength and power, our results revealed notable differences between VBT and PBT in sprint and COD performance. Specifically, 20-m sprint times improved by 3.7% (ES = 1.30) in the VBT group compared to 1.6% (ES = 0.51) in the PBT group, with the VBT group showing significantly superior improvement. Similarly, the agility T-test revealed greater performance enhancements in the VBT group (−3.2%, ES = 0.54) than in the PBT group (−1.5%, ES = 0.54). These findings support previous reports indicating velocity-based training’s potential to enhance short-distance speed and agility more effectively than traditional fixed-load training (Banyard et al., 2021; Pareja-Blanco et al., 2017). Several underlying mechanisms could explain the superior sprint and agility improvements observed in the VBT group. First, VBT enables real-time load adjustments based on velocity feedback, ensuring each session provides an optimal neuromuscular stimulus without inducing excessive fatigue (Weakley et al., 2021). This precise intensity regulation likely maintains higher quality training sessions, consistently promoting favorable neural adaptations critical for maximal acceleration and rapid direction changes. Furthermore, instant velocity feedback may encourage maximal voluntary effort and intent during training repetiticagoons, which has been linked to improved motor unit recruitment and firing rate, enhancing explosive power generation crucial for sprint and agility performance (Gonzalez-Badillo et al., 2014). Additionally, improved sprint and COD performance may be partly attributed to enhanced neuromuscular efficiency, including increased stiffness of the muscle-tendon complex and optimized stretch-shortening cycle (SSC) function. Regular training at higher intended velocities, as prescribed by VBT, may preferentially enhance the athlete’s ability to rapidly transition between eccentric and concentric muscle actions, translating directly to improved acceleration and agility outcomes (Loturco et al., 2016; Suchomel et al., 2016).

While the present study, along with a significant body of existing research, demonstrates advantages of VBT over PBT in enhancing strength, speed, and agility, it is essential to interpret these results cautiously and objectively. Not all existing studies have consistently shown VBT to be superior to PBT under all conditions. Specifically, differences between VBT and PBT in improving strength and explosive power appear minimal among individuals with limited or no training experience, likely due to heightened responsiveness of beginners to structured training stimuli, irrespective of the training method employed (Orange et al., 2020; Mann et al., 2010). Furthermore, some studies have suggested that PBT could even yield comparable or superior results when the training focus is on muscle hypertrophy or muscular endurance, primarily because traditional percentage-based loading allows sustained high-volume and high-load training, generating greater mechanical stimuli that favor muscle growth (Ahtiainen et al., 2003; Schoenfeld et al., 2017). Moreover, the choice between VBT and PBT in practical training environments is influenced by factors beyond physiological effectiveness alone, including logistical constraints and management complexity. Although VBT provides individualized, real-time load adjustments responsive to daily athlete readiness, its broader implementation can be limited due to higher costs associated with equipment, technical expertise required from coaches, and complexities in session management, particularly within large groups (Weakley et al., 2021). Conversely, PBT offers simplicity, standardized procedures, and broader acceptance by coaches and athletes, making it particularly practical in grassroots or resource-constrained settings.

Therefore, strength and conditioning professionals should carefully consider factors such as training objectives, athlete characteristics, logistical constraints, team size, and available resources when choosing between VBT and PBT, rather than assuming universal superiority of either method.

## 5 Limitations and additional considerations

Although this study provides valuable insights, several limitations should be noted. Firstly, the relatively small sample size (n = 20) may restrict the generalizability of the findings; future studies should include larger cohorts to confirm the observed differences between VBT and PBT. Secondly, the intervention period was limited to 6 weeks; longer-duration training programs could yield further insights into chronic adaptations and sustainability of training effects. Additionally, this study involved only trained male individuals, limiting extrapolation to other populations, such as female athletes or less experienced lifters. Future research should consider these populations and possibly integrate additional performance metrics, such as neuromuscular assessments or muscle hypertrophy evaluations, to provide a more comprehensive understanding of the training adaptations induced by VBT *versus* PBT.

## 6 Practical applications

From a practical standpoint, strength and conditioning coaches may consider incorporating VBT methods into their resistance training programs to optimize performance outcomes. Specifically, VBT allows individualized and dynamic load adjustments, ensuring that athletes consistently train at intended intensities while minimizing excessive fatigue. Coaches and practitioners may use real-time velocity feedback (e.g., linear position transducers) to monitor athletes’ performance and rapidly adjust training loads, potentially enhancing maximal strength, sprinting speed, and agility more effectively than traditional fixed-percentage methods.

## 7 Conclusion

The present study indicates that both velocity-based training (VBT) and traditional percentage-based training (PBT) protocols effectively improve lower-body power, maximal strength, sprinting speed, and agility performance in trained individuals. However, VBT appears to provide superior gains specifically in maximal strength (1RM back squat), linear sprint (20-m sprint), and COD (T-test) performance compared to PBT, likely due to the individualized load adjustments and fatigue management enabled by real-time velocity feedback. Thus, incorporating velocity monitoring into resistance training may optimize training adaptations more effectively than traditional fixed-percentage methods, highlighting its potential value in strength and conditioning practice.

## Data Availability

The raw data supporting the conclusions of this article will be made available by the authors, without undue reservation.
